# Ghrelin Induces Clu^+^ Revival Stem Cells and Regenerates Lgr5^+^ Stem Cells via the Vagus Nerve to Mitigate Gastrointestinal Acute Radiation Syndrome

**DOI:** 10.3390/ijms27156781

**Published:** 2026-07-29

**Authors:** Fangming Zhang, Hui Jin, Gaifeng Ma, Asha Jacob, Ping Wang, Max Brenner

**Affiliations:** 1 Center for Immunology and Inflammation, The Feinstein Institutes for Medical Research, Manhasset, NY 11030, USA; 2 Departments of Surgery and Molecular Medicine, Zucker School of Medicine at Hofstra/Northwell, Manhasset, NY 11030, USA

**Keywords:** ghrelin, vagus nerve, gastrointestinal acute radiation syndrome, intestinal stem cells, Lgr5, clusterin, partial body irradiation, radiation medical countermeasure, neuroenteric regulation

## Abstract

Gastrointestinal acute radiation syndrome (GI-ARS) is a deadly consequence of radiation exposure. We hypothesized that the peptide ghrelin is enteroprotective after radiation injury, and that ghrelin promotes intestinal stem cell regeneration via the vagus nerve. We subjected mice to 12-Gy partial body irradiation (PBI) with 5% bone marrow sparing. Some mice were vagotomized prior to PBI. We then injected the mice with human ghrelin (6 nmol/mouse) or vehicle at 24, 48, and 72 h post-irradiation, and collected blood and tissues at 96 h. PBI caused an 80% reduction in plasma citrulline, 32% shorter villi, 59% fewer crypts, a 14-fold increase in TUNEL^+^ cells, a 9-fold increase in intestinal permeability (FD4), and 4- to 30-fold increases in bacterial translocation (16S rRNA) to the liver and mesentery. Ghrelin significantly improved all these parameters. Remarkably, vagotomy attenuated ghrelin’s protective effects by 22–58%. Mechanistically, ghrelin increased proliferating crypt cells by 2.3-fold, Lgr5^+^ active stem cells by 2.6-fold, Clu^+^ revival stem cells by 3.3-fold (immunofluorescence), and Clu mRNA by 1.6-fold compared to PBI alone, and all these effects were significantly diminished by vagotomy. Thus, ghrelin mitigates GI-ARS through vagus nerve-dependent activation of Clu^+^ revival stem cells and Lgr5^+^ stem cells, identifying a novel vagal-dependent neuroenteric pathway that regulates intestinal crypt regeneration after radiation injury.

## 1. Introduction

Nuclear accidents and natural disasters, radiological terrorism, and the nuclear arsenal pose a persistent threat of mass radiation exposure to civilian and military populations [1,2,3,4,5]. Depending on the dose absorbed, radiation exposure causes three staggering acute radiation syndromes (ARS) [6]. Hematopoietic syndrome (H-ARS) occurs after doses as low as 2–5 Gy and is characterized by pancytopenia [7,8,9]. Gastrointestinal syndrome (GI-ARS), which develops after exposure to doses of 6 Gy or more, consists of the destruction of the intestinal epithelium, resulting in mucosal barrier dysfunction, bacterial translocation, sepsis, and elevated mortality [10]. Neurovascular syndrome occurs after extremely high doses (>30 Gy) and is invariably lethal [11]. While several FDA-approved medical countermeasures (MCMs) exist for H-ARS [12], no MCMs have been approved for GI-ARS, despite its high morbidity and mortality.

The intestinal epithelium is the most rapidly renewing tissue in the body, with a complete turnover cycle of approximately 5 days [13]. This renewal is driven by Leucine-rich repeat-containing G-protein coupled receptor 5 (Lgr5) stem cells residing at the base of intestinal crypts [14]. Due to their highly proliferative nature, Lgr5^+^ stem cells are exquisitely sensitive to radiation-induced DNA damage and cell death. The radiation-induced loss of Lgr5^+^ stem cells interrupts the basal-to-apical migration of epithelial cells, leading to villous denudation, barrier dysfunction, and ultimately GI-ARS [10,15,16]. When Lgr5^+^ stem cells are depleted by radiation, a distinct population of quiescent reserve stem cells—recently identified as clusterin (Clu) revival stem cells—undergoes transient expansion and dedifferentiation to reconstitute the Lgr5^+^ stem cell pool and regenerate the intestinal epithelium [17,18]. Thus, the coordinated activity of Clu^+^ revival stem cells and Lgr5^+^ stem cells is essential for intestinal recovery after radiation injury.

Ghrelin is a 28-amino acid acylated peptide hormone predominantly secreted by X/A-like endocrine cells in the gastric mucosa [19]. Beyond its well-characterized roles in appetite regulation, growth hormone secretion, and energy homeostasis, ghrelin exerts pleiotropic beneficial effects on gastrointestinal, cardiovascular, neuroendocrine, and immune systems [19,20]. Ghrelin readily crosses the blood–brain barrier (BBB) [21]. In the central nervous system (CNS) ghrelin activates hypothalamic ghrelin receptors (ghrR, a.k.a. growth hormone secretagogue receptor 1a, GHSR-1a) to promote gastric acid secretion and motility and inhibit intestinal epithelial cell apoptosis via stimulation of dorsal vagal complex neurons, the vagus nerve, parasympathetic postganglionic (intestinal wall) neurons, and the enteric nervous system (ENS), all of which are cholinergic [19,22,23,24,25]. Importantly, ghrelin is also a trophic factor for the gastrointestinal mucosa [26,27,28].

Prior studies from our group and others have demonstrated ghrelin’s enteroprotective effects in preclinical models of total and partial body irradiation (TBI, PBI) [29,30,31,32]. Our studies of ghrelin in sepsis and gut ischemia–reperfusion established that ghrelin is enteroprotective and acts primarily through a centrally mediated neuroenteric mechanism involving the vagus nerve [33,34,35,36,37]. We have also discovered that administration of ghrelin induces Clu^+^ revival stem cells and promotes the recovery and expansion of the Lgr5^+^ stem cells in the intestinal crypts of mice with GI-ARS following PBI [29]. These observations suggest that regulation of intestinal stem cell activation represents a mechanistically central pathway through which ghrelin promotes post-radiation recovery. However, whether the vagus nerve regulates intestinal stem cell biology has not been previously demonstrated. Furthermore, whether ghrelin’s mitigation of GI-ARS involves vagus nerve-dependent regulation of intestinal stem cells has never been directly investigated. This question represents an important gap in our understanding of neuroenteric communication and intestinal regeneration.

In this study, we tested the hypothesis that ghrelin mitigates GI-ARS via the vagus nerve by promoting intestinal crypt regeneration through the activation of both Clu^+^ revival stem cells and Lgr5^+^ stem cells. The primary objective was to establish the necessity of vagal signaling for ghrelin’s protection; the secondary objective was to characterize which intestinal stem cell populations mediate this vagus nerve-dependent mechanism. Using a mouse model of 12 Gy PBI with subdiaphragmatic vagotomy, we systematically evaluated the effects of vagotomy on ghrelin-mediated improvements in intestinal integrity, barrier function, apoptosis, proliferation, and stem cell responses. Our findings reveal a previously unrecognized vagal-dependent mechanism through which the brain regulates intestinal stem cell activity after radiation injury, with important implications for the development of MCMs for GI-ARS, the management of radiation therapy side effects, and the treatment of other intestinal conditions involving stem cell dysfunction.

## 2. Results

### 2.1. Vagotomy Reverses Ghrelin’s Protective Effects on Intestinal Integrity After PBI

**Figure 1:**
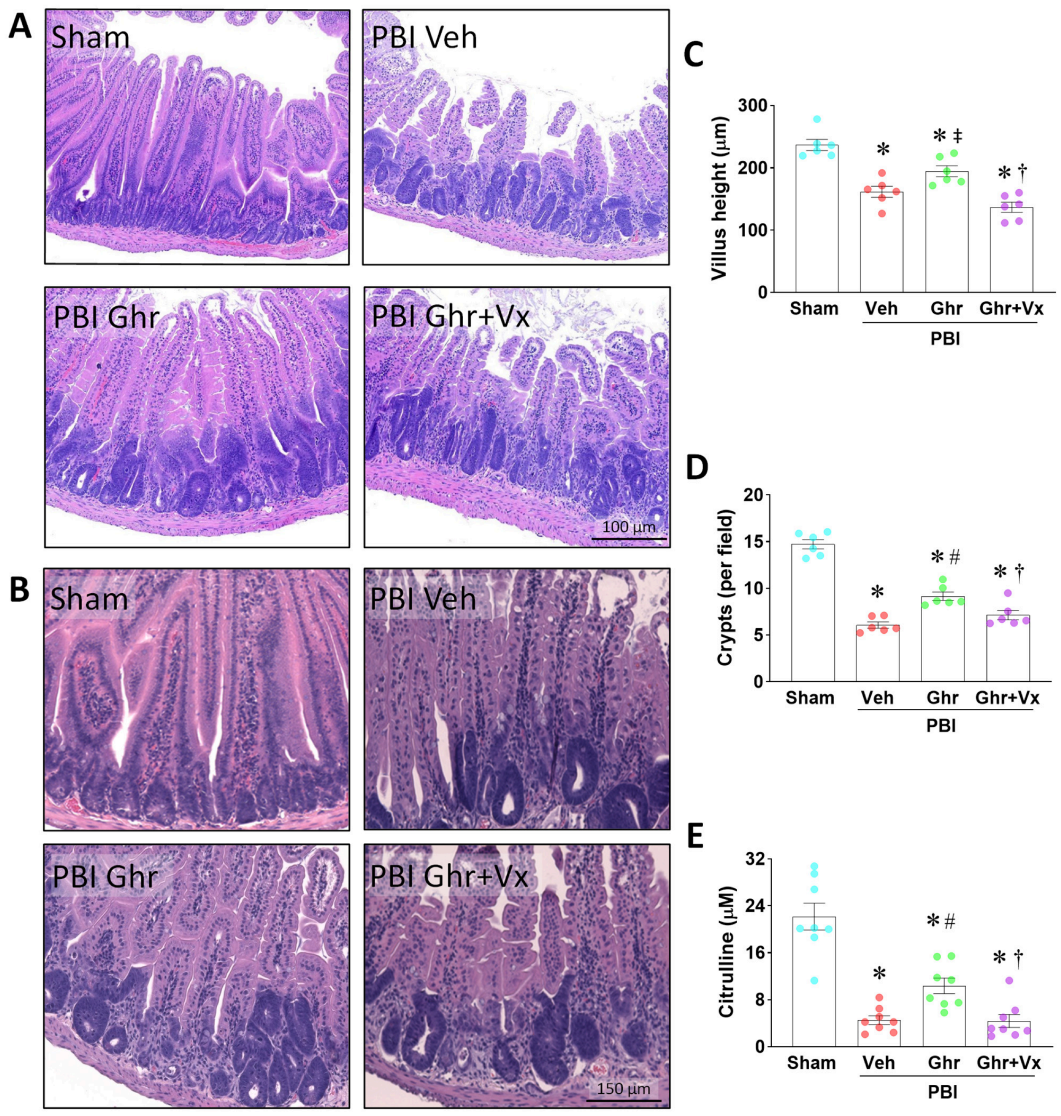
Vagotomy Reverses Ghrelin’s Protective Effects on Intestinal Integrity after Partial Body Irradiation. (**A**,**B**) Representative hematoxylin and eosin (H&E)-stained jejunal cross-sections from sham, PBI vehicle-treated (PBI Veh), PBI + ghrelin-treated (PBI Ghr), and PBI + ghrelin + vagotomized (PBI Ghr + Vx) mice showing villus architecture and crypt morphology. Day 4 post-irradiation, H&E, magnification indicated. (**C**) PBI caused a 32% reduction in villus height compared to sham, which was partially restored by ghrelin treatment (20% improvement); however, vagotomy significantly attenuated this benefit by 30%. (**D**) PBI reduced crypt counts by 59%, ghrelin increased crypts by 51% over PBI alone, and vagotomy reduced this ghrelin-mediated improvement by 22%. Crypts per field. (**E**) PBI caused an 80% reduction in citrulline, ghrelin treatment increased levels by 128%, and vagotomy abolished this protective effect by 58%. Day 4 plasma; ELISA. ANOVA; * *p* < 0.05 vs. Sham, ^#^ *p* < 0.05 vs. PBI Veh, ^‡^ *p* = 0.07 vs. PBI Ghr, ^†^ *p* < 0.05 vs. PBI Ghr (n = 6 mice/group).

To investigate whether ghrelin’s protection against radiation-induced intestinal injury requires an intact vagus nerve, we subjected mice to subdiaphragmatic vagotomy (Vx) or sham surgery prior to 12 Gy PBI, followed by treatment with ghrelin (6 nmol/mouse, subcutaneously, at 24, 48, and 72 h post-irradiation) or vehicle. Jejunal tissues were collected on day 4 for histological analysis (Figure 1).

Irradiation (PBI Veh) caused a significant reduction in jejunal villus height compared to sham controls, representing a 32% decrease (Figure 1A–C). Ghrelin treatment (PBI Ghr) partially restored villus height, representing a 20% improvement over PBI Veh. Importantly, vagotomy (Vx) significantly attenuated ghrelin’s beneficial effect, with the PBI Ghr + Vx group showing a 30% reduction in villus height compared to the PBI Ghr group (Figure 1C). Notably, villus height in the PBI Ghr + Vx group was even shorter than in the PBI-only group, suggesting that vagotomy not only abrogated ghrelin’s benefit but may have exacerbated injury in the context of irradiation.

PBI markedly reduced crypt density, with crypt counts declining by 59% compared to sham (Figure 1A,B,D). Ghrelin treatment significantly increased crypt numbers in PBI, Ghr mice, representing a 51% increase over PBI Veh. Vagotomy significantly attenuated this improvement, resulting in a 22% decrease in crypt counts in the PBI Ghr + Vx group relative to the PBI Ghr group (Figure 1D).

Citrulline, a nonprotein amino acid synthesized by enterocytes, serves as a validated biomarker of enterocyte mass and radiation-induced small intestinal damage [38,39]. PBI Veh caused an 80% reduction in plasma citrulline levels compared to sham (Figure 1E). Ghrelin treatment significantly preserved citrulline levels (10.3 ± 1.3, representing a 128% increase over PBI Veh. Vagotomy abolished this protective effect, with citrulline levels in the PBI Ghr + Vx group declining by 58% compared to the PBI Ghr group and approaching the levels observed in PBI Veh mice (Figure 1E). Taken together, these results demonstrate that ghrelin attenuates radiation-induced intestinal injury and that the vagus nerve is essential for this protection.

### 2.2. Vagotomy Eliminates Ghrelin’s Anti-Apoptotic Effect on the Irradiated Intestine

**Figure 2:**
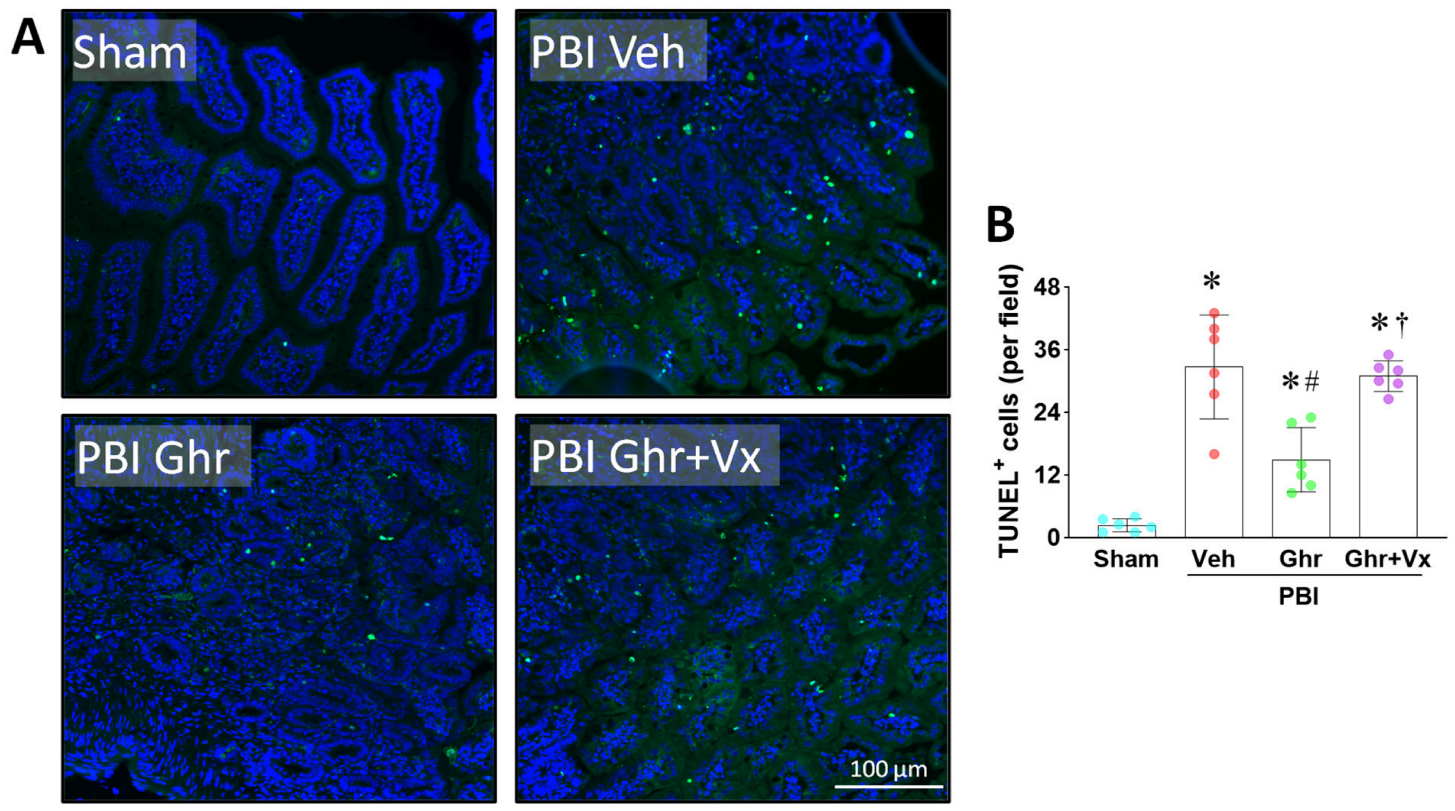
Vagotomy Eliminates Ghrelin’s Anti-Apoptotic Effect on the Irradiated Intestine. (**A**) Representative TUNEL-stained jejunal sections showing apoptotic cells (bright green nuclei) in intestinal crypts across experimental groups. TUNEL, counterstained with DAPI (blue) to visualize nuclei. (**B**) PBI caused a 14-fold increase in apoptotic cells compared to sham controls; ghrelin reduced apoptotic cells by 54%; vagotomy reversed this anti-apoptotic effect by 2.1-fold compared to the PBI Ghr group, approaching levels in PBI Veh mice. ANOVA; * *p* < 0.05 vs. Sham, ^#^ *p* < 0.05 vs. PBI Veh, ^†^ *p* < 0.05 vs. PBI Ghr (n = 6 mice/group).

Radiation-induced apoptosis of crypt cells is a critical early event in the pathogenesis of GI-ARS [15,16]. To assess whether ghrelin attenuates crypt cell apoptosis and whether this effect depends on the vagus nerve, we performed TUNEL staining on jejunal sections (Figure 2).

PBI dramatically increased the number of TUNEL-positive apoptotic cells in intestinal crypts, with a 14-fold increase compared to sham controls (Figure 2A,B). Ghrelin treatment significantly reduced apoptotic cell numbers by 54% (Figure 2B). Vagotomy markedly attenuated ghrelin’s anti-apoptotic effect, with TUNEL-positive cells increasing by 2.1-fold in the PBI Ghr + Vx group compared to the PBI Ghr group (Figure 2B), approaching the levels observed in the PBI Veh group. These findings indicate that ghrelin mitigates radiation-induced intestinal cell apoptosis through a vagus nerve-dependent mechanism.

### 2.3. Vagotomy Attenuates Ghrelin’s Protection of Intestinal Barrier Function After PBI

**Figure 3:**
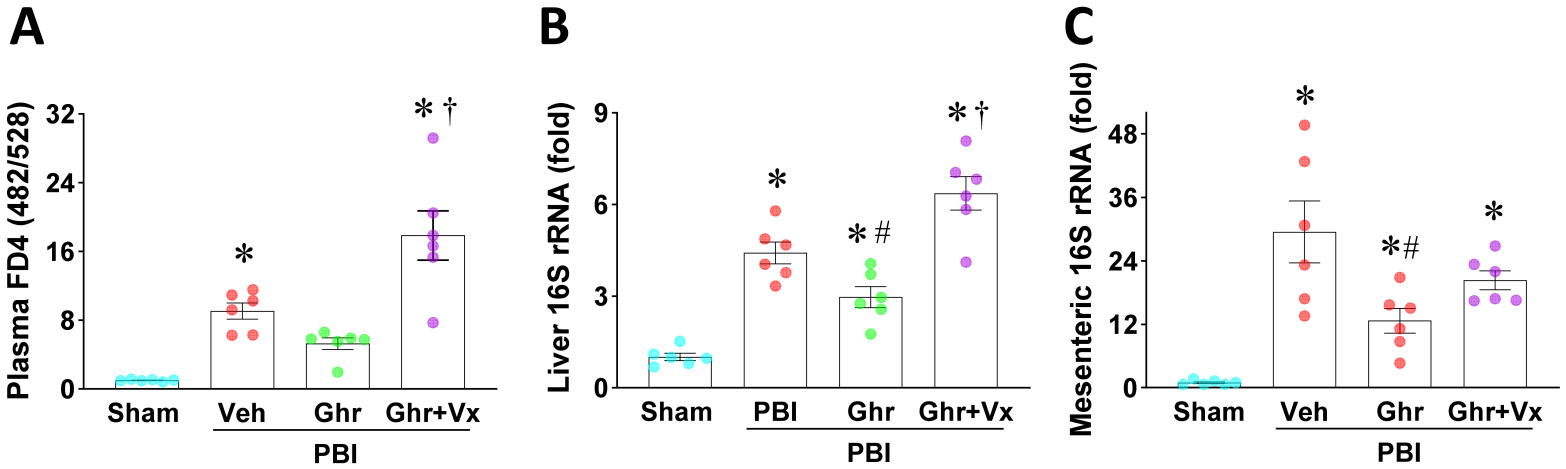
Vagotomy Abolishes Ghrelin’s Protection of Intestinal Barrier Function after Partial Body Irradiation. (**A**) PBI caused a 9.1-fold increase in FD4 permeability; ghrelin reduced levels by 42%; vagotomy reversed this protection 3.4-fold and resulted in intestinal permeability nearly twice that of PBI Veh mice. Plasma fluorescence 60 min after intraluminal injection of 4 kDa FITC-dextran (25 mg/mL). (**B**) PBI increased hepatic 16S rRNA 4.4-fold; ghrelin reduced bacterial translocation by 33%; vagotomy reversed this effect 2.1-fold. (**C**) PBI increased mesenteric 16S rRNA 30-fold; ghrelin reduced levels by 57%; vagotomy attenuated this reduction by 60% compared to the PBI Ghr group, though not statistically significant between PBI Ghr + Vx and PBI Ghr groups. Day 4 liver and mesenteric samples, qPCR. ANOVA; * *p* < 0.05 vs. Sham, ^#^ *p* < 0.05 vs. PBI Veh, ^†^ *p* < 0.05 vs. PBI Ghr (n = 6 mice/group).

Radiation-induced damage to the intestinal epithelium leads to increased intestinal permeability—commonly termed “leaky gut”—which facilitates the translocation of bacteria to the intestinal wall, draining lymph nodes, and blood, causing sepsis and multi-organ failure [16,40]. We evaluated intestinal barrier function using two complementary approaches: measurement of plasma 4 kDa FITC-dextran (FD4) fluorescence following intraluminal administration and quantification of bacterial translocation by 16S rRNA qPCR in liver (hematogenic route) and mesenteric tissues (contiguous and lymphatic routes) (Figure 3).

Intestinal permeability (FD4): To avoid possible artifactual increases due to impaired gastric emptying following vagotomy, FD4 was administered by direct intraluminal injection into a ligated segment of jejunum. PBI caused a 9.1-fold increase in plasma FD4 fluorescence compared to sham (Figure 3A), indicating severe barrier dysfunction. Ghrelin treatment reduced plasma FD4 levels by 42% (Figure 3A). Vagotomy completely reversed this protective effect, with FD4 levels increasing 3.4-fold in the PBI Ghr + Vx group compared to the PBI Ghr group (Figure 3A). Strikingly, intestinal permeability in the PBI Ghr + Vx group was nearly twice that observed in the PBI Veh group, suggesting that vagotomy in the setting of irradiation not only completely abrogates the beneficial effect of ghrelin but may further compound barrier injury.

Bacterial translocation—liver: PBI increased hepatic 16S rRNA expression by 4.4-fold relative to sham (Figure 3B), consistent with bacterial translocation from the gut. Ghrelin reduced hepatic 16S rRNA expression by 33%. Vagotomy reversed this effect, with the PBI Ghr + Vx group showing a 114% increase in hepatic 16S rRNA compared to the PBI Ghr group (Figure 3B).

Bacterial translocation—mesentery: Similarly, PBI increased mesenteric 16S rRNA expression by 30-fold relative to sham (Figure 3C). Ghrelin treatment reduced mesenteric 16S rRNA by 57%. Vagotomy attenuated this reduction, with the PBI Ghr + Vx group exhibiting a 60% increase over the PBI Ghr group (Figure 3C). It should be noted that mesenteric lymph nodes were not isolated from surrounding mesenteric tissue due to technical challenges, leading to detection of high levels of 16S rRNA with increased variability, which may explain the absence of a statistically significant difference between the PBI Ghr + Vx and PBI Ghr groups in mesenteric 16S rRNA expression.

Collectively, these results demonstrate that ghrelin significantly improves intestinal barrier function and reduces bacterial translocation after PBI, and that the vagus nerve is required for these protective effects.

### 2.4. Vagotomy Reduces Ghrelin-Enhanced Intestinal Crypt Proliferation After PBI

**Figure 4:**
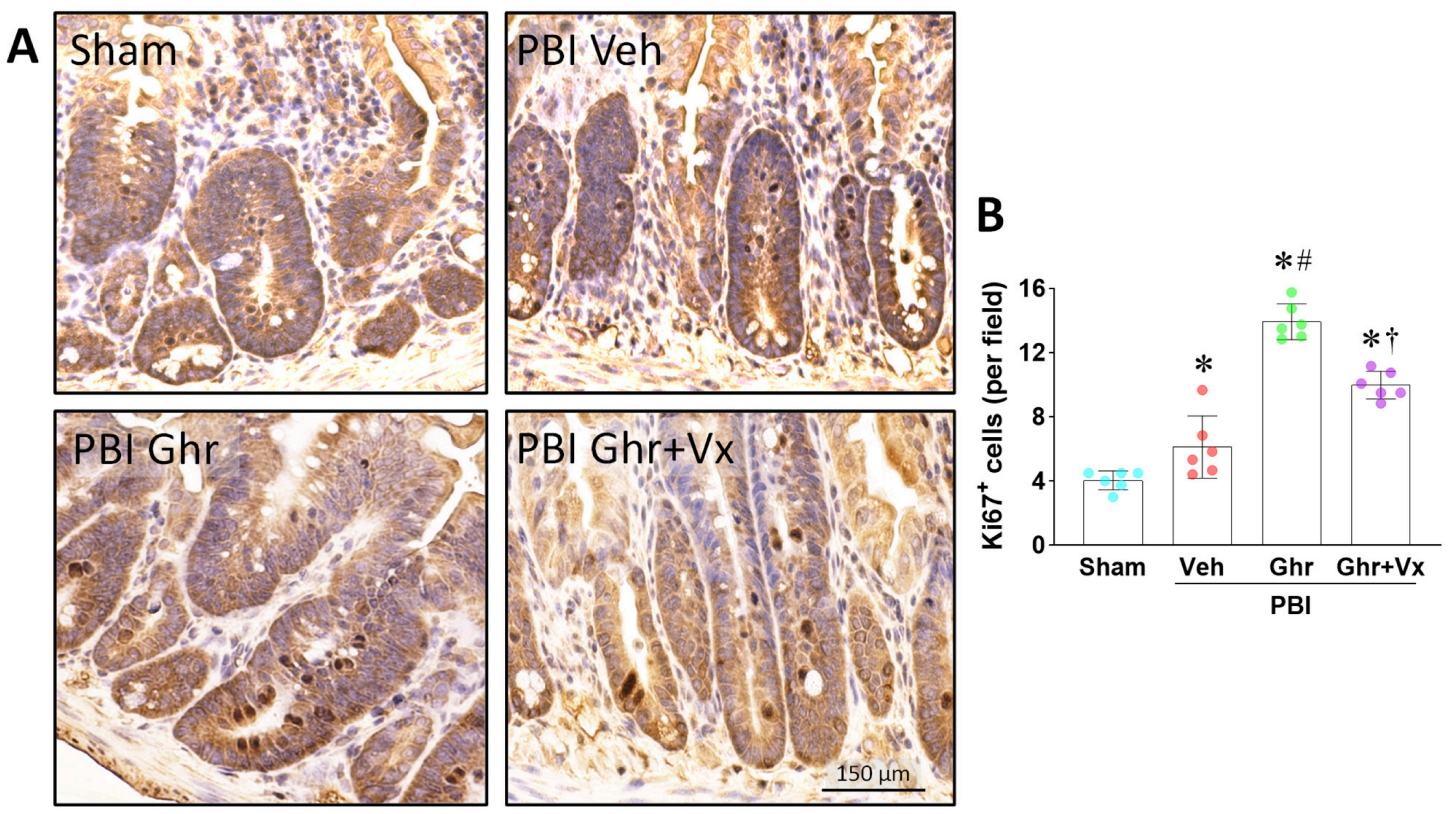
Vagotomy Reduces Ghrelin-Enhanced Intestinal Crypt Proliferation after Partial Body Irradiation. (**A**) Representative immunohistochemical staining and quantification of Ki67^+^ cells (cell with brown-stained nuclei). (**B**) PBI modestly increased Ki67^+^ cells by 1.5-fold over sham; ghrelin dramatically amplified this response 2.3-fold over PBI Veh and 3.4-fold over sham; vagotomy significantly attenuated ghrelin’s pro-proliferative effect by 28%. Day 4 jejunal sections incubated overnight at 4 °C with rabbit anti-Ki67 primary antibody (1:500); HRP-conjugated secondary antibody and visualized with DAB; Ki67^+^ nuclei quantified using ImageJ. ANOVA; * *p* < 0.05 vs. Sham, ^#^ *p* < 0.05 vs. PBI Veh, ^†^ *p* < 0.05 vs. PBI Ghr (n = 6 mice/group).

Intestinal recovery after radiation injury requires the proliferative expansion of surviving crypt cells to reconstitute the epithelium. Ki67 is a nuclear protein expressed exclusively during active phases of the cell cycle (G1, S, G2, and M) and serves as a well-established marker of proliferative activity [41]. We assessed Ki67 expression in jejunal crypts by immunohistochemistry (Figure 4).

PBI modestly increased Ki67^+^ cells in the crypts compared to sham (Figure 4A,B), consistent with partial initiation of a proliferative response at day 4 post-irradiation. Ghrelin treatment dramatically amplified this response, with Ki67^+^ cells increasing by 2.3-fold compared to PBI Veh and 3.4-fold compared to sham (Figure 4B). Vagotomy significantly attenuated ghrelin’s pro-proliferative effect, with Ki67^+^ cells decreasing by 28% in the PBI Ghr + Vx group compared to the PBI Ghr group (Figure 4B). These results indicate that ghrelin enhances the proliferative response of intestinal crypt cells after PBI, and that this effect is partly dependent on the vagus nerve.

### 2.5. Vagotomy Abolishes Ghrelin-Mediated Enhancement of Intestinal Stem Cells After PBI

**Figure 5:**
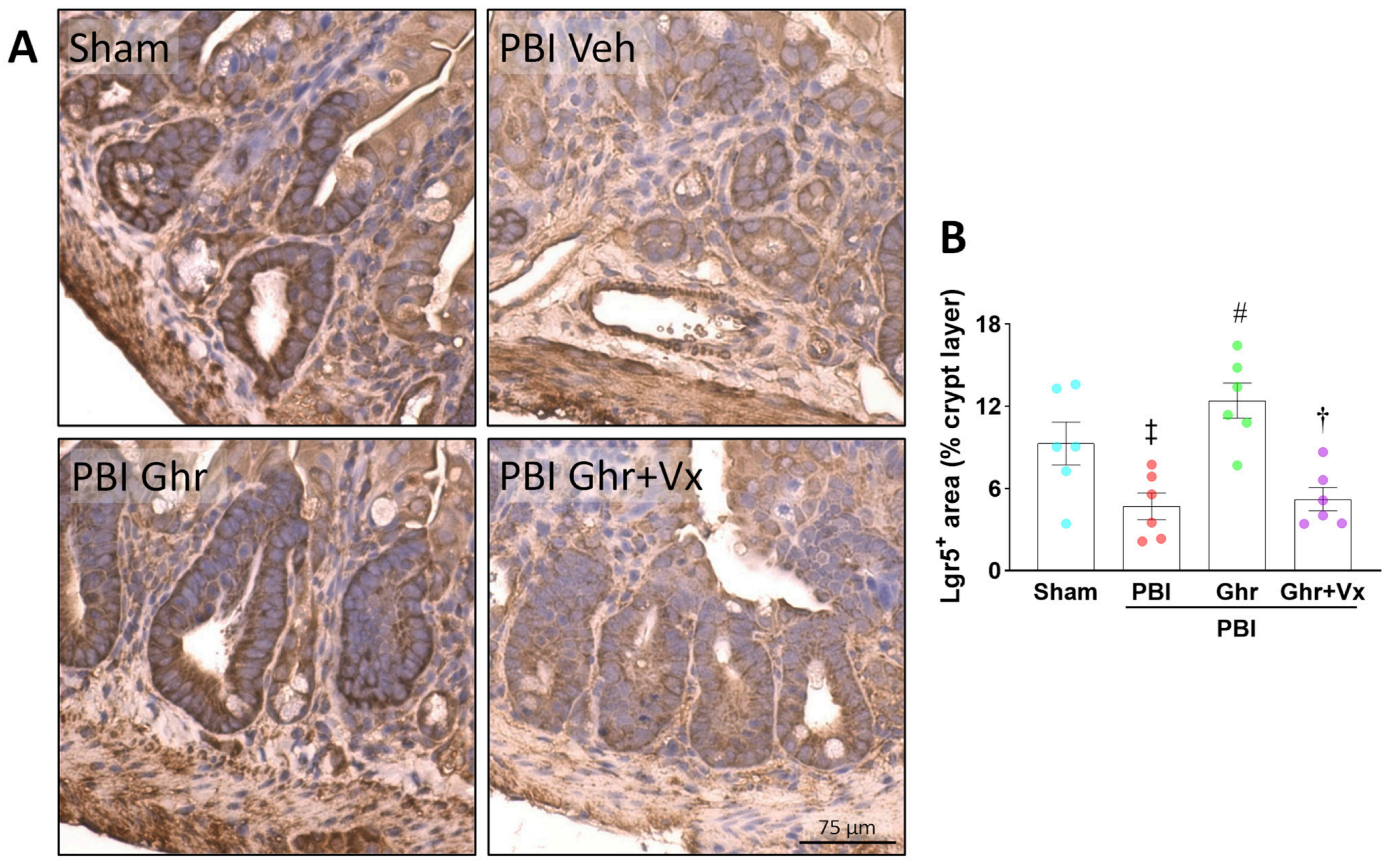
Vagotomy Abolishes Ghrelin-Mediated Enhancement of Lgr5^+^ Intestinal Stem Cells after Partial Body Irradiation. (**A**) Representative immunohistochemical staining of Lgr5^+^ intestinal stem cells (brown-stained cells) at the crypt base across experimental groups. (**B**) PBI caused a 49% reduction in Lgr5^+^ cells; ghrelin restored and exceeded sham levels by 2.6-fold; vagotomy dramatically reversed this effect by 58%, reducing Lgr5^+^ cells to levels comparable to PBI Veh. Day 4 jejunal sections incubated overnight at 4 °C with rabbit anti-Lgr5 primary antibody (1:250); HRP-conjugated secondary antibody and visualized with DAB; nuclei counterstained with hematoxylin; Lgr5^+^ cells quantified using ImageJ. ANOVA; ^‡^ *p* = 0.07 vs. Sham, ^#^ *p* < 0.05 vs. PBI Veh, ^†^ *p* < 0.05 vs. PBI Ghr (n = 6 mice/group).

The turnover of the intestinal epithelium is driven by fast-cycling self-renewing multipotent crypt-base columnar cells at the bottom of the crypts that are characterized by surface expression of Lgr5 [42]. Due to their fast-cycling nature, Lgr5^+^ stem cells are very sensitive to radiation-induced cell death. Therefore, to determine whether ghrelin’s enteroprotective effects include the regulation of intestinal stem cells, we examined intestinal crypt Lgr5^+^ stem cells by immunohistochemistry (Figure 5).

PBI caused a 49% reduction in Lgr5^+^ cells compared to sham (Figure 5A,B), consistent with radiation-induced Lgr5^+^ stem cell depletion. Ghrelin treatment restored and exceeded sham levels, with Lgr5^+^ cells reaching 12.4 ± 1.3—a 2.6-fold increase over PBI Veh (Figure 5B). Vagotomy dramatically reduced this effect by 58%, with the PBI Ghr + Vx group showing as few Lgr5^+^ cells as PBI Veh (Figure 5B). These data demonstrate that ghrelin promotes the recovery of Lgr5^+^ stem cells after radiation injury through a vagus nerve-dependent mechanism.

### 2.6. Vagotomy Reduces Ghrelin-Mediated Promotion of Intestinal Revival Stem Cells After PBI

**Figure 6:**
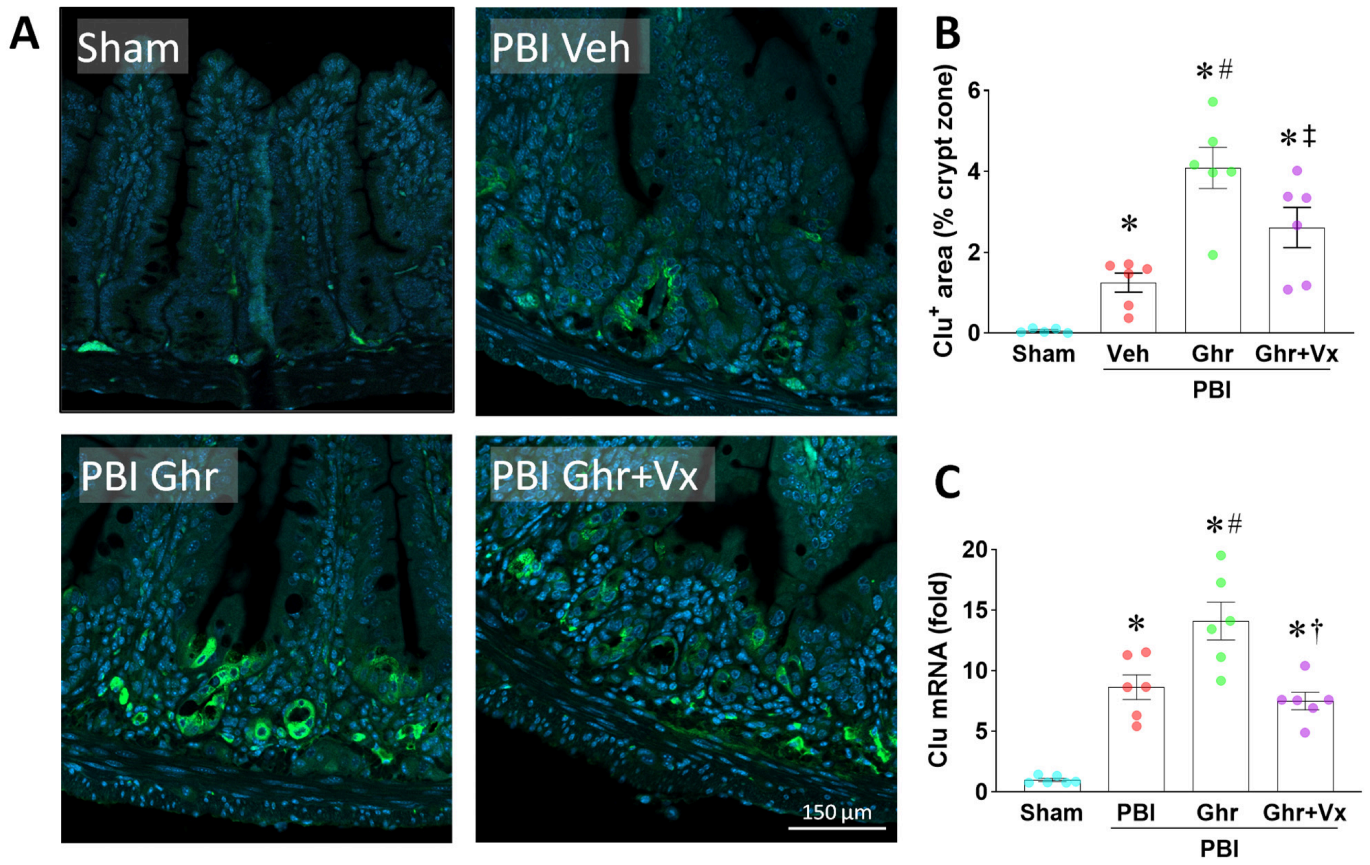
Vagotomy Reduces Ghrelin-Mediated Promotion of Clusterin^+^ Revival Stem Cells after Partial Body Irradiation. (**A**) Representative immunofluorescence images of Clusterin (Clu)^+^ revival stem cells (green fluorescence) in jejunal crypts. Day 4 jejunal sections incubated with rabbit anti-clusterin primary antibody (1:250); donkey anti-rabbit Alexa Fluor 488 secondary antibody (1:500); and counterstained with DAPI (blue) to visualize nuclei. (**B**) No Clu^+^ cells were detected in sham crypts; PBI induced Clu^+^ cell appearance; ghrelin further increased Clu^+^ signal by 3.3-fold over PBI alone; vagotomy reduced Clu^+^ signal by 36% compared to PBI Ghr group. Semi-quantitative analysis of Clu^+^ cell immunoreactive signal intensity as percentage of crypt zone area; ImageJ. (**C**) PBI induced 8.6-fold upregulation of Clu mRNA; ghrelin further increased Clu mRNA 1.6-fold to 14.1-fold over sham (63% increase over PBI alone); vagotomy reduced Clu mRNA by 47% compared to PBI Ghr group, falling below PBI-only levels. qPCR. ANOVA; * *p* < 0.05 vs. Sham, ^#^ *p* < 0.05 vs. PBI Veh, ^†^ *p* < 0.05 vs. PBI Ghr, ^‡^ *p* = 0.06 vs. PBI Ghr (n = 6 mice/group).

When Lgr5^+^ stem cells die, they must be regenerated by otherwise quiescent and thus radioresistant Clu^+^ revival stem cells typically located at crypt position +4 (4 cells from the bottom of the crypt) [18]. Since intestinal epithelial recovery after radiation injury is a function of crypt stem cells, we evaluated whether ghrelin’s enteroprotective effects involve the regulation of Clu^+^ revival stem cells (Figure 6).

We examined Clu expression at both the mRNA and protein levels. In line with the literature [18], immunofluorescence staining revealed no Clu^+^ revival stem cells in the intestinal crypts of sham mice, and increased Clu^+^ cells in the crypts following PBI, with the greatest increase observed in the PBI Ghr group (Figure 6A,B). Vagotomy reduced Clu^+^ cell numbers by 36% in the PBI Ghr + Vx group compared to the PBI Ghr group (Figure 6B). These findings were further supported with jejunal Clu mRNA expression. PBI induced a significant upregulation of Clu mRNA compared to sham (Figure 6C), consistent with the activation of revival stem cells after radiation injury. Ghrelin treatment produced the greatest increase in Clu mRNA levels, reaching 14.1-fold relative to sham—a 63% increase over PBI alone (Figure 6C). Vagotomy significantly reduced Clu expression, with the PBI Ghr + Vx group showing a 47% decrease compared to the PBI Ghr group (Figure 6C). Notably, Clu mRNA levels in the PBI Ghr + Vx group were even lower than in the PBI-only group, suggesting that vagotomy not only blocked ghrelin’s effect but may have also impaired the endogenous revival stem cell response.

## 3. Discussion

In the present study, we demonstrate for the first time that ghrelin mitigates GI-ARS through a vagus nerve-dependent mechanism that activates both Clu^+^ revival stem cells and Lgr5^+^ stem cells. Using a mouse model of 12 Gy PBI with subdiaphragmatic vagotomy, we show that ghrelin preserves intestinal structural integrity, reduces crypt cell apoptosis, improves intestinal barrier function, enhances crypt proliferation, and promotes intestinal stem cell recovery—and that all of these protective effects are significantly attenuated or abolished by vagotomy. These findings establish a novel neuroenteric pathway by which the central nervous system, via the vagus nerve, regulates intestinal crypt stem cell responses after radiation injury.

Our results confirm and extend previous findings that ghrelin is protective against radiation-induced intestinal injury [28,29,31,37,43]. PBI caused severe intestinal damage, as evidenced by a 32% reduction in villus height, 59% loss of crypts, and 80% decline in plasma citrulline—a validated biomarker of functional enterocyte mass [38,39]. Ghrelin significantly improved all these parameters, consistent with prior studies in TBI models and in PBI models [29,31]. The novel finding of this study is that vagotomy substantially reversed ghrelin’s beneficial effects on all three measures of intestinal integrity. The reduction in citrulline levels following vagotomy was particularly striking (58% decrease compared to PBI Ghr), indicating that the preservation of enterocyte mass by ghrelin is predominantly mediated through vagal signaling.

The observation that villus height in vagotomized mice treated with ghrelin (PBI Ghr + Vx group) was shorter than in vehicle-treated PBI mice also warrants consideration. This finding may reflect the combined detrimental effects of vagotomy and irradiation on the intestinal epithelium. Vagotomy eliminates tonic parasympathetic input to the gastrointestinal tract, which is known to maintain basal crypt proliferation and epithelial homeostasis [44,45]. The loss of this tonic input, superimposed on radiation-induced stem cell depletion, may result in a more severe epithelial injury than radiation alone.

Radiation-induced apoptosis of crypt cells, particularly stem cells, is a critical early event that correlates with the severity of GI-ARS [16]. We found that ghrelin reduced TUNEL-positive apoptotic cells in the crypts by 54% compared to PBI alone, while vagotomy reversed this effect, increasing apoptotic cells 2.1-fold compared to the ghrelin-treated group. These findings are consistent with previous observations in TBI rats, where ghrelin reduced crypt apoptosis by 62% and shifted the Bcl-xL/Bax ratio toward anti-apoptotic signaling [31]. The anti-apoptotic mechanism of ghrelin in this context likely involves direct cholinergic signaling from the vagus nerve (or via the ENS) to crypt cells. Muscarinic receptor activation has been shown to decrease apoptosis through multiple pathways, including PI3K/Akt signaling, upregulation of anti-apoptotic Bcl-2 family members, and inhibition of caspase activation [46]. Moreover, activation of the α7 nicotinic acetylcholine receptor (α7nAChR) has similarly been shown to suppress apoptosis in intestinal epithelial cells [47]. Given that intestinal crypt cells have been shown to express cholinergic receptors [45], it is possible that ghrelin’s anti-apoptotic effects on crypt stem cells are mediated by direct cholinergic stimulation from the ENS.

Intestinal barrier dysfunction is the hallmark of GI-ARS and the proximate cause of bacterial translocation, sepsis, and death [16]. We assessed barrier function using two complementary approaches: direct measurement of paracellular permeability using intraluminal FD4 and quantification of bacterial translocation by 16S rRNA qPCR. Both assays demonstrated that ghrelin significantly improved barrier function after PBI and that vagotomy reversed these effects. The magnitude of the increase in FD4 permeability in the PBI Ghr + Vx group (3.4-fold over PBI Ghr) was notably larger than the vagotomy-mediated reversal of other parameters. This may reflect the additive effects of vagotomy-induced impairment of mucosal defense mechanisms—including reduced mucus secretion, diminished epithelial tight junction integrity, and impaired mucosal blood flow—on top of the radiation-induced barrier damage [48]. Vagus nerve stimulation has been shown to protect intestinal tight junctions by maintaining the expression of claudins, occludin, and ZO-1 through α7nAChR signaling [47]. The loss of this protective input through vagotomy would be expected to exacerbate barrier dysfunction. The 16S rRNA data confirmed that bacterial translocation to the liver and mesentery was significantly reduced by ghrelin and increased by vagotomy. The lack of statistical significance in the mesenteric data may relate to the technical limitation of not isolating mesenteric lymph nodes from surrounding mesenteric fat and connective tissue, which would dilute the bacterial signal.

The regenerative capacity of the intestinal epithelium after radiation injury depends on the proliferative expansion of surviving crypt cells. We found that ghrelin dramatically enhanced Ki67^+^ proliferating cells in the crypts (3.4-fold over sham; 2.3-fold over PBI Veh), and that vagotomy reduced this effect by 28%. The observation that Ki67 levels in the PBI Veh group showed only a modest increase (1.5-fold over sham) at day 4 is consistent with the known cell cycle arrest that occurs after radiation-induced DNA damage, followed by gradual recovery [49]. Ghrelin appears to accelerate and amplify this proliferative recovery.

The vagus nerve’s role in stimulating crypt proliferation is supported by studies demonstrating that cholinergic signaling promotes intestinal epithelial cell proliferation through muscarinic and nicotinic receptors [44,50,51,52,53,54,55]. The M1 muscarinic receptor, in particular, has been shown to specifically regulate proliferation in the crypt stem cell compartment [50]. Nicotinic receptor signaling through Hippo and Notch pathways also contributes to intestinal stem cell function [54]. Our finding that vagotomy reduced ghrelin-enhanced crypt proliferation supports the model in which ghrelin acts centrally to enhance vagal-cholinergic input not only to the gut, which was already known [24], but specifically to the intestinal crypts, thereby promoting the proliferative expansion of stem and progenitor cells.

Perhaps the most important finding of this study is the demonstration that ghrelin promotes the activation of both Lgr5^+^ stem cells and Clu^+^ revival stem cells after PBI through the vagus nerve. Lgr5^+^ stem cells are the actively cycling stem cells responsible for ongoing epithelial renewal [14,56]. PBI caused a 49% reduction in Lgr5^+^ cells, consistent with their well-known radiosensitivity. Ghrelin treatment not only restored Lgr5^+^ cells to sham levels but exceeded them by 34%, further confirming our previous discovery that ghrelin promotes both the recovery and expansion of the Lgr5^+^ stem cell pool [29]. The Lgr5^+^ cell increase likely reflects the upstream activation of Clu^+^ revival stem cells, which dedifferentiate to replenish the Lgr5^+^ pool [18], as well as direct effects on Lgr5^+^ cell survival and proliferation [28]. Vagotomy significantly attenuated this effect by 58%, reducing Lgr5^+^ cells in the PBI Ghr + Vx group to levels comparable to PBI Veh. This was the largest vagotomy-induced reversal observed across all parameters, underscoring the critical dependence of ghrelin’s stem cell-promoting effects on vagal signaling.

Clu^+^ revival stem cells are quiescent reserve stem cells that are activated specifically in response to severe injury [17,18]. After radiation-induced death of Lgr5^+^ stem cells, Clu^+^ cells undergo p53-dependent expansion and dedifferentiation to reconstitute the Lgr5^+^ stem cell pool [17,18,57,58]. In our study, PBI induced a significant upregulation of Clu at both the mRNA (8.7-fold) and protein (21-fold) levels, consistent with activation of the revival stem cell program. Ghrelin further amplified this response, increasing Clu mRNA by 1.6-fold and Clu protein by 3.3-fold over PBI alone. These results further confirm our previous discovery that ghrelin promotes Clu^+^ revival stem cell expansion [29]. Moreover, our findings that ghrelin increases Clu expression concurrently with Lgr5^+^ cell recovery are consistent with ghrelin activating this established regenerative program. However, as lineage tracing was not performed in the present study, we cannot exclude the possibility that the observed Lgr5^+^ cell recovery also reflects direct effects of ghrelin on surviving Lgr5^+^ stem cells [28]. Vagotomy reduced Clu expression by 36–47%, with Clu mRNA in the PBI Ghr + Vx group falling below PBI Veh levels. This finding is particularly striking because it suggests that vagal input not only mediates ghrelin’s enhancement of Clu^+^ cell activation but may also contribute to the basal revival stem cell response after radiation injury. We speculate that tonic vagal parasympathetic activity provides a permissive signal for the activation of the Clu^+^ revival stem cell program, and that ghrelin amplifies this signal through enhanced vagal output.

Based on our findings and the existing literature, we propose the following model for ghrelin’s mitigation of GI-ARS. Systemically administered ghrelin crosses the BBB [21] and activates GhrR in hypothalamic neurons and dorsal vagal complex efferent neurons [20]. This is proposed to stimulate efferent vagal output through preganglionic cholinergic neurons [24], which synapse onto postganglionic parasympathetic neurons in the intestinal wall to innervate intestinal crypts directly or via the myoenteric and submucosal plexuses (the ENS), which are also cholinergic [45]. Cholinergic stimulation may then promote: 1. survival of crypt cells by suppressing apoptosis; 2. activation and expansion of Clu^+^ revival stem cells; 3. dedifferentiation of Clu^+^ cells to replenish the Lgr5^+^ stem cell pool; and 4. proliferation and expansion of Lgr5^+^ stem cells. Even though further studies are needed to directly characterize the downstream neural and cellular mediators within this pathway, the clear end result is regeneration of the intestinal epithelium, restoring barrier function and preventing bacterial translocation.

These findings have direct relevance to the development of MCMs for GI-ARS, an unmet medical need [10]. Human ghrelin has several features that make it an attractive candidate MCM. First, it has been evaluated in over 100 Phase I clinical trials with an excellent safety profile. The most common adverse event was transient flushing, which led to discontinuation in only 3 of 939 participants [59]. Second, ghrelin can be synthesized in large quantities under cGMP (current good manufacturing practice) conditions, making it suitable for mass emergency deployment. Third, ghrelin can be administered subcutaneously, facilitating field deployment. Fourth, our dose–response studies have demonstrated that ghrelin dose-dependently improved the 30-day survival of PBI mice from 25% to 78% [29]. The present study provides mechanistic insight into ghrelin’s efficacy by demonstrating that it promotes intestinal stem cell-mediated regeneration through a neuroenteric pathway. This mechanistic understanding is important for the development of ghrelin as an MCM under the FDA’s Animal Rule, which requires a thorough understanding of the mechanism of action of drugs approved based on animal efficacy data [60]. Moreover, this vagus nerve-dependent stem cell activation mechanism is expected to also extend to other clinically relevant irradiation scenarios such as pelvic and abdominal radiotherapy.

Our findings have implications beyond radiation biology. The discovery that the brain, via the vagus nerve, may regulate intestinal stem cell activity represents an important advance in our understanding of brain–gut communication. While the vagus nerve is well known to regulate gastrointestinal motility, secretion, and immunity [24,61,62,63], its role in regulating stem cell biology has not been previously demonstrated. This proposed neuroenteric stem cell regulatory mechanism may be relevant to a wide range of clinical conditions involving intestinal stem cell dysfunction. Vagal regulation of intestinal stem cells may also contribute to mucosal healing in the contexts of actinic enteropathy [40], chemotherapy [57], intestinal ischemia/reperfusion injury [36], and inflammatory bowel disease (IBD) [64,65]. Conversely, suppression of vagal regulation may be beneficial to suppress colorectal cancer cell proliferation, radio resistance, and chemoresistance [66,67,68].

Noteworthy limitations of this study should be noted. Although sex differences in radiation sensitivity have been documented, the mechanisms underlying these differences remain poorly characterized, and the directionality varies by tissue and radiation dose. Moreover, ghrelin responsiveness exhibits sex-dependent variation mediated by estrogen signaling and cyclical hormonal fluctuations. Given the mechanistic focus of this study—establishing whether the vagus nerve mediates ghrelin’s protection through intestinal stem cell activation—male mice were selected to minimize hormonal confounding and maintain biological homogeneity. This design enables rigorous investigation of neuroenteric signaling pathways; however, it limits generalizability and necessitates future validation in female mice to assess whether this protective mechanism is sex-independent or requires hormonal modulation. The vagotomy procedure itself may have confounding effects on intestinal physiology, including impaired gut motility and changes in mucosal blood flow. We attempted to minimize confounding from impaired gastric emptying by using intraluminal rather than oral administration of FD4 for permeability assessment. However, pyloroplasty was not performed following vagotomy because mice survived for only four days, and the resulting variable gastric distention may have introduced some variability. While we did not employ a formal verification method such as electrophysiological assessment of vagal conduction or quantification of parasympathetic neural markers, multiple lines of indirect evidence support the effectiveness of the procedure: 1. vagotomized mice consistently developed reduced peristalsis, a well-recognized consequence of vagal denervation, as indicated by mild gastric distension, and 2. our laboratory has extensive experience with subdiaphragmatic vagotomy in both rats and mice [36,37,69,70,71,72,73], and the procedure was performed by a highly experienced surgeon using established surgical protocols. Both the anterior and posterior trunks of the vagus nerve were carefully identified under surgical microscopy and completely transected. This surgical technique has been validated in prior studies demonstrating that vagotomy produces characteristic effects on immune regulation and inflammatory cytokine production consistent with loss of vagal cholinergic signaling [37,69]. Importantly, incomplete vagotomy would bias the results toward showing no difference between vagotomized and non-vagotomized groups, which is the opposite of what we observed. Nevertheless, future studies should include formal verification methods to definitively confirm complete vagal denervation. Third, Lgr5 immunohistochemistry was challenging to quantify due to weak signal intensity and non-specific background staining. Although the observed differences between groups were large and consistent with all other measured parameters, these results should be interpreted with caution. Nevertheless, this issue does not invalidate the overall conclusion of the study. However, future studies should employ complementary methods such as Lgr5-reporter mice or flow cytometry to more accurately quantify Lgr5^+^ stem cell numbers. All analyses were performed at day 4 post-irradiation, which represents the nadir of intestinal injury but captures only a single snapshot of the regenerative process. Studies incorporating multiple time points are needed to fully characterize the dynamics of ghrelin-mediated intestinal stem cell regeneration. In addition, the study did not include a PBI + vagotomy group without ghrelin treatment. However, this does not substantially limit interpretation of our primary findings. The experimental comparison directly tests whether the vagus nerve mediates ghrelin’s effects on intestinal stem cell recovery. Since ghrelin’s mechanism of action via hypothalamic activation and vagal signaling is well-established for gastrointestinal motility and secretion [19,20,24], it is mechanistically plausible that the identical neuroenteric pathway regulates intestinal stem cell biology. The observation that vagotomy attenuates ghrelin’s effects across all measured parameters—intestinal integrity, barrier function, apoptosis, proliferation, and stem cell recovery—directly supports the conclusion that the vagus nerve is necessary for ghrelin’s protective mechanism. Nevertheless, a PBI + vagotomy vehicle control group would provide additional phenotypic data regarding vagotomy’s independent contribution to radiation-induced intestinal dysfunction and could be included in future studies. We acknowledge that amplification efficiencies were not formally assessed by standard curve analysis in this study. However, the validity of our conclusions is supported by multiple lines of evidence: 1. the observed fold-changes in expression (1.6-fold to 14.1-fold) are substantially larger than typical variations introduced by amplification efficiency deviations; 2. results were highly consistent across biological replicates within each group; 3. mRNA expression patterns (qPCR) were independently corroborated at the protein level by immunofluorescence for clusterin, demonstrating that transcriptional changes translate to functional protein-level changes; 4. expression patterns conform to expected biology (Clu absent in sham, induced by PBI, amplified by ghrelin, reduced by vagotomy); and 5. the 2^−ΔΔCT^ method was applied uniformly across all experimental groups, minimizing potential systematic bias from amplification efficiency variations. Finally, the mesenteric bacterial translocation data did not achieve statistical significance between PBI Ghr and PGI Ghr + Vx groups, possibly because mesenteric lymph nodes were not isolated from surrounding tissue. Future studies should use microdissected mesenteric lymph nodes for more precise assessment.

In conclusion, we demonstrate that ghrelin mitigates GI-ARS by promoting the activation of Clu^+^ revival stem cells and Lgr5^+^ stem cells via a neuroenteric mechanism involving the vagus nerve. The ghrelin/vagus nerve/intestinal stem cell axis represents a previously unrecognized mechanism of brain–gut communication whose downstream mediators remain to be fully elucidated, with important implications for the development of MCMs for GI-ARS. Modulation of this pathway may also be valuable for the management of radiotherapy-induced intestinal injury, the treatment of other conditions involving intestinal stem cell dysfunction, and even suppression of proliferation of colorectal carcinoma cells.

## 4. Materials and Methods

### 4.1. Experimental Animals

Healthy male C57BL/6 mice (9–12 weeks of age) were purchased from Charles River Laboratories (Wilmington, MA, USA) and housed at the Feinstein Institutes Animal Facility. Animals underwent a minimum 1-week acclimation period prior to any procedures. Mice were housed in groups of 4–5 per cage (523 cm^2^ of floor space) at a temperature of 18–26 °C with 30–70% relative humidity and a 12-h light cycle. Cages were provided with environmental enrichment including nesting material and a cardboard shelter. Water and standard laboratory chow were provided ad libitum. All experiments were conducted in accordance with the National Institutes of Health guidelines for the use of experimental animals and were approved by the Institutional Animal Care and Use Committee (IACUC) of the Feinstein Institutes for Medical Research (Protocol #24-1093).

### 4.2. Animal Study Design

A priori sample size calculation (n = 6 mice/group) was performed assuming a two-tailed *t*-test with α = 0.05, power = 0.80, and expected effect size of 1.4 standard deviations based on our previous studies [29,31]. This sample size was sufficient to detect meaningful differences in primary endpoints (citrulline levels, crypt counts). C57BL/6 mice (n = 24; 9–12 weeks old) were randomly allocated to four experimental groups: 1. Sham—sham surgery with vehicle treatment; 2. PBI + Vehicle—partial body irradiation with saline injection; 3. PBI + Ghrelin—partial body irradiation with human ghrelin treatment; and 4. PBI + Ghrelin + Vagotomy (PBI Ghr + Vx)—subdiaphragmatic vagotomy performed prior to PBI, followed by ghrelin treatment. Randomization was performed at the cage level using a simple randomization scheme (assigning cages to groups before any procedures began). Mice had the same age and weight and did not require baseline stratification. Investigators conducting outcome assessments (histological analysis, immunohistochemistry) were blinded to group assignment during image analysis and data quantification. Unblinding occurred only after data analysis was complete. The experimental unit was the individual mouse. This study design allowed systematic evaluation of the vagus nerve’s requirement for ghrelin’s protective effects across multiple parameters of intestinal integrity and stem cell function. Post-operative analgesia following vagotomy was provided with buprenorphine (0.1 mg/kg, subcutaneous) immediately after surgery. Mice were monitored daily for signs of pain, infection, or distress. Humane endpoints were established a priori and included: 1. inability to ambulate, 2. Body Condition Score of 2 or less, 3. Grimace score of 2, 4. hunched posture, 5. weight loss exceeding 20% of baseline, 6. bloody stools; or 7. signs of peritonitis. All mice survived to the planned euthanasia timepoint (day 4). No unexpected adverse events were recorded. No animals were excluded from any analysis.

### 4.3. Partial Body Irradiation (PBI)

During irradiation, unanesthetized mice were briefly restrained and placed in fitted clear acrylic containers. The bilateral hind extremities (fibula, tibia, and feet) were shielded using lead tubes, thereby sparing approximately 5% of the bone marrow [29,74,75]. Mice were then exposed to a 12 Gy dose of PBI using an X-ray irradiator (X-RAD 320; PXi, North Branford, CT, USA) with filter 2 (1.5 mm Al + 2.5 mm Cu + 0.75 Sn) at a dose rate of ~1 Gy/min, operating at 320 kVp and 12.5 mA, with a source-to-surface distance (SSD) of 50 cm. Immediately after irradiation, mice were returned to their cages. Sham mice underwent identical handling and placement procedures but were not exposed to radiation.

### 4.4. Subdiaphragmatic Vagotomy (Vx)

All surgical procedures were performed under aseptic conditions with sterilized instruments. Before irradiation, mice in the PBI Ghr + Vx group were anesthetized with 2% inhaled isoflurane and positioned supine. Vx was performed according to our previously described method [69,73]. Briefly, the abdominal area was shaved and disinfected, and a 2.5 cm midline incision was made below the xiphoid process to expose the stomach. The liver lobes were gently retracted to allow visualization of the esophagus, and the dorsal and ventral branches of the vagus nerve were carefully identified by surgical microscopy and transected just below the diaphragm under surgical microscopy. Pyloroplasty was not performed following vagotomy, as survival was limited to four days. Immediately after surgery, mice were administered subcutaneous injections of 0.1 mg/kg buprenorphine and 1 mL saline. Variable degrees of gastric distention were observed among the mice on day 4 post-vagotomy; however, no mortality occurred prior to sacrifice.

### 4.5. Ghrelin Administration

O-n-octanoylated (acylated, active) human ghrelin (CPC Scientific, Rocklin, CA, USA) was synthesized under current good manufacturing practice (cGMP). Mice were randomly assigned to four groups: sham, PBI (vehicle), PBI + ghrelin (PBI Ghr), and PBI + ghrelin + vagotomy (PBI Ghr + Vx). Mice in the PBI Ghr + Vx and PBI Ghr groups received three subcutaneous doses of human ghrelin (6 nmol/mouse in 100 μL of normal saline) administered at 24, 48, and 72 h after irradiation based on a dosing regimen previously shown by our group to be effective in mitigating radiation injury [29,32]. Mice in the PBI group received 100 μL of normal saline subcutaneously.

### 4.6. Tissue Collection

Mice were euthanized by CO_2_ inhalation 4 days after radiation or combined radiation and vagotomy. The abdominal cavity was opened, and venous blood was collected via puncture of the inferior vena cava. The jejunum (10 cm segment of the small intestine beginning 5 cm distal to the ligament of Treitz) was harvested. A mid-portion ~2 cm segment of the jejunum was fixed in 10% formaldehyde for histological analysis, and the remaining jejunum, liver samples and mesentery samples were flash-frozen in liquid nitrogen and stored at −80 °C for protein and gene expression analyses.

### 4.7. Assessment of Intestinal Permeability

A separate cohort of mice was used to assess intestinal permeability. Due to reduced gastric peristalsis following vagotomy, intestinal permeability was evaluated by intraluminal administration of FD4, based on the method described by Ewing et al. [76] with minor modifications. Briefly, at 4 days post-irradiation and vagotomy, mice were anesthetized, and a 2 cm midline abdominal incision was made. A 10 cm segment of the small intestine was isolated, beginning 5 cm distal to the ligament of Treitz. The segment was ligated at both ends using 4-0 sutures. Subsequently, 0.3 mL of 4 kDa FITC-dextran (25 mg/mL in PBS; Sigma-Aldrich, St. Louis, MO, USA) was injected into the lumen from the proximal end using a 30-gauge needle. The distal end near the injection site was additionally ligated to prevent leakage. The abdominal incision was closed, and mice were returned to their cages. Sixty minutes after injection, heparinized blood was collected, and plasma was obtained by centrifugation at 3000× *g* for 5 min at 4 °C. Plasma samples were diluted 25-fold with normal saline, and FITC-dextran fluorescence was measured using a fluorescence spectrophotometer (Synergy HT, BioTek Instruments, Winooski, VT, USA) at excitation 485 nm and emission 528 nm. Results were normalized to the sham group.

### 4.8. Plasma Citrulline ELISA

Plasma citrulline levels were measured using a mouse citrulline ELISA kit (MyBioSource, San Diego, CA, USA) according to the manufacturer’s instructions.

### 4.9. Histological Analysis

Intestinal samples were fixed in formalin, embedded in paraffin, and sectioned at 5 μm thickness. Sections were stained with hematoxylin and eosin (H&E). Villus height and crypt counts were measured under a light microscope at 100× magnification by investigators blinded to the group of origin. For histological quantification, villus height and crypt depth were performed across 5 high-power fields (HPF) containing well-oriented villi per mouse. For each HPF, villus height was measured as the distance from the villus base to the apex (average of 3–5 villi per HPF), and crypt counts were obtained by counting all crypts visible within the HPF. Values from all 5 HPF were averaged to generate a single data point per animal.

### 4.10. TUNEL Staining

Tissue sections were stained using a TUNEL assay kit (Roche Diagnostics, Indianapolis, IN, USA) according to the manufacturer’s instructions. Briefly, sections were deparaffinized, permeabilized with Triton X-100, and incubated with the TUNEL reaction mixture containing TdT enzyme and labeled nucleotides for 60 min at 37 °C in a humidified dark chamber. After washing, TUNEL-positive cells were quantified in five randomly selected fields per section under a fluorescence microscope by investigators blinded to the group of origin.

### 4.11. Immunohistochemistry (Ki67 and Lgr5)

Deparaffinized sections were subjected to antigen retrieval with citrate buffer (pH 6.0) in a microwave oven, permeabilized with 0.1% Triton X-100, and blocked with 5% normal serum for 1 h at room temperature. Sections were incubated overnight at 4 °C with primary antibodies against Ki67 (rabbit anti-Ki67, 1:500, 28074-1-AP, Proteintech, Rosemont, IL, USA) or Lgr5 (rabbit anti-Lgr5, 1:250, MA5-32108, Invitrogen Carlsbad, CA, USA). Both antibodies were obtained from established commercial vendors with published validation for mouse tissue and the intended applications. Ki67 was validated for immunohistochemistry of proliferating cells in mouse intestinal tissue; Lgr5 was validated for immunohistochemistry in mouse small intestine. Sections were then incubated with HRP-conjugated horse anti-mouse/rabbit IgG secondary antibody (PK-7200, Vector Laboratories, Newark, CA, USA) and visualized using 3,3′-diaminobenzidine (DAB; SK-4100, Vector Laboratories). Nuclei were counterstained with hematoxylin. Ki67-positive nuclei per field were quantified in a blinded manner using ImageJ Version 1.54p. Lgr5-positive cells were quantified using ImageJ. Ki67 signal was restricted to the proliferative compartment of intestinal crypts, consistent with its established distribution.

### 4.12. Immunofluorescence (Clusterin)

Tissue sections were incubated overnight at 4 °C with rabbit anti-clusterin (1:250, 23159S, Cell Signaling Technology, Danvers, MA, USA). The antibody was obtained from an established commercial vendor with published validation for immunofluorescence in mouse tissues. Sections were then incubated with donkey anti-rabbit Alexa Fluor 488 secondary antibody (1:500, A21206, Invitrogen) for 1 h at room temperature. Slides were counterstained with DAPI, mounted using ProLong Gold (Invitrogen), and imaged on a Zeiss LSM confocal microscope (Zeiss, White Plains, NY, USA). Clu-positive signal intensity was quantified using ImageJ to generate a semi-quantitative immunoreactive score. Due to the diffuse cytoplasmic staining pattern of clusterin, Clu^+^ cells were quantified by measuring the Clu-immunoreactive signal area as a percentage of total crypt zone area rather than by direct cell counts, as individual cell boundaries were difficult to delineate with this marker. Clusterin immunofluorescence showed no signal in sham intestinal crypts and was induced specifically following irradiation, consistent with known biology that Clu^+^ revival stem cells are quiescent under homeostasis and activated only after severe injury. Clu protein expression by immunofluorescence was independently corroborated by Clu mRNA expression assessed by qPCR across all experimental groups, providing cross-modal validation of the immunostaining results.

### 4.13. Real-Time Quantitative RT-PCR

Total RNA was extracted from intestinal, liver, and mesentery tissues using TRIzol reagent (Invitrogen). cDNA was synthesized using M-MLV reverse transcriptase (Applied Biosystems, Foster City, CA, USA). PCR reactions were performed in 25 μL volumes containing 0.08 μM of each primer, cDNA template, DEPC-treated water, and SYBR Green Master Mix (Applied Biosystems). Amplification and analysis were conducted using the StepOnePlus Real-Time PCR System (Applied Biosystems). Mouse β-actin mRNA served as the internal control, and relative expression was calculated using the 2^−ΔΔCT^ method. Results are presented as fold changes relative to sham-treated tissues. Primer sequences: 16S rRNA forward, 5′-AGAGTTGATCMTGGCTCAG-3′; reverse, 5′-TACGGYTACCTTGTTACGACTT-3′; Clu forward, 5′-CAGCTGGCTAACCTCACACA-3′; reverse, 5′-CTATCTCATTCCGCACGGCT-3′; β-actin forward, 5′-CGTGAAAAGATGACCCAGATCA-3′; reverse, 5′-TGGTACGACCAGAGGCATACAG-3′.

### 4.14. Statistical Analysis

Data were analyzed using GraphPad Prism software (Version 10.4.1) (GraphPad Software, San Diego, CA, USA) and are presented in the text and figures as mean ± SEM. Prior to statistical test selection, data were assessed for normality of distribution using the Shapiro–Wilk test and for homogeneity of variances using the Brown–Forsythe test and Bartlett’s test. All datasets passed these assumption tests (*p* > 0.05), confirming that the data met the requirements for parametric statistical testing. Two-way comparisons used Student’s *t*-test and multi-way comparisons used one-way ANOVA with Student–Newman–Keuls post-hoc testing. A *p*-value ≤ 0.05 was considered statistically significant.

## Data Availability

The data generated in the present study may be requested from the corresponding authors.

## Abbreviations

The following abbreviations are used in this manuscript:

ARS	Acute radiation syndrome
BBB	Blood–brain barrier
BrdU	Bromodeoxyuridine
cGMP	Current good manufacturing practice
ChAT	Choline acetyltransferase
Clu	Clusterin
CNS	Central nervous system
CO_2_	Carbon dioxide
DAB	3,3′-Diaminobenzidine
DAPI	4′,6-Diamidino-2-phenylindole
DEPC	Diethylpyrocarbonate
DMF	Dose-modifying factor
DNA	Deoxyribonucleic acid
ELISA	Enzyme-linked immunosorbent assay
ENS	Enteric nervous system
FD4	Fluorescein isothiocyanate–conjugated 4-kDa dextran
FDA	Food and Drug Administration
FdUrd	Fluorodeoxyuridine
FITC	Fluorescein isothiocyanate
Ghr	Ghrelin
GhrR	Ghrelin receptor
GHSR-1a	Growth hormone secretagogue receptor 1a
GI-ARS	Gastrointestinal acute radiation syndrome
Gy	Gray
H&E	Hematoxylin and eosin
H-ARS	Hematopoietic acute radiation syndrome
HRP	Horseradish peroxidase
IACUC	Institutional Animal Care and Use Committee
IBD	Inflammatory bowel disease
IF	Immunofluorescence
IHC	Immunohistochemistry
IVC	Inferior vena cava
kDa	Kilodalton
kV	Kilovolt
Lgr5	Leucine-rich repeat-containing G-protein coupled receptor 5
M1, M3	Muscarinic receptor subtypes 1 and 3
mA	Milliampere
MCM	Medical countermeasure
MLN	Mesenteric lymph node
mRNA	Messenger ribonucleic acid
Nβ1	Nicotinic β1 receptor
NIH	National Institutes of Health
nAChR (α7nAChR)	(Alpha-7) Nicotinic acetylcholine receptor
PBI	Partial body irradiation
PBS	Phosphate-buffered saline
PCR	Polymerase chain reaction
PG	PBI + ghrelin group
qPCR	Quantitative polymerase chain reaction
RCI	Radiation combined injury
RNA	Ribonucleic acid
rRNA	Ribosomal ribonucleic acid
RT-PCR	Reverse transcription polymerase chain reaction
sc	Subcutaneous
SDV	Subdiaphragmatic vagotomy
SEM	Standard error of the mean
SSD	Source-to-surface distance
TBI	Total body irradiation
TUNEL	Terminal deoxynucleotidyl transferase–mediated dUTP nick-end labeling
Veh	Vehicle
VNS	Vagus nerve stimulation
Vx	Vagotomy
ZO-1	Zonula occludens-1
α7nAChR	Alpha-7 nicotinic acetylcholine receptor
2^−ΔΔCT^	Delta–delta cycle threshold method
